# Colchicum in Acute Rheumatism

**Published:** 1881-08

**Authors:** P. J. Farnsworth

**Affiliations:** Clinton, Iowa


### COLCHICUM IN ACUTE RHEUMATISM.

By P. J. FARNSWORTH, M.D., of Clinton, Iowa.

In the early days of my practice I went out with the old doctor to see a patient, six or seven miles from home. We found a large, strong man, who had been taken rather suddenly, with a high fever, and severe pains in his joints, in his knees and ankles especially, which were also somewhat swollen, a thick coat on his tongue. The slightest move was agony to him, and a jar of the bed gave him pain. The doctor at once pronounced it a case of inflammatory rheumatism.

Opening the capacious medicine chest which the doctor of the old time carried, he first gave him some powders of calomol and jalap, five grains of the first to ten of the last; this was inevitable; such a tongue demanded it; then from the store of everything, from castor oil to cantharides, he drew out some solid extract of colchicum. The woman of the house procured a half pint bottle, and the doctor guessed off half drachm of the extract and put it into the bottle, with a little tincture of cinnamon. It was filled with water, and directions given to administer a tablespoonful every three hours, beginning one hour after one of the powders. Some Dover's powders (pulv. ipecac. c.) say of five grains each, were also left, to be given when the medicine operated. The limbs were ordered wrapped in cloths wrung out of hot water.

The next morning I was directed to make the patient a visit. The woman met me at the door, with concern in her face, and the inquiry in regard to taking the medicine in the bottle. The man said a tablespoonful every three hours, the woman insisted that it was three tablespoonfuls every hour. She being the nurse, and persistent about it, had had her own way, and had faithfully followed for some time her version of the directions. At the third hour nausea had ensued, but the medicine was continued; by the fourth, vomiting and purging commenced. A Dover's powder was given and the dose from the bottle omitted until the sixth hour, when the vomiting and purging was so severe that the woman became alarmed, and had given the cathartic powder that was left over, which not being retained, she had resorted to peppermint tea and spice bags over the stomach, for relief.

When I arrived the patient was almost in a state of collapse, nearly pulseless, with feeble respiration, and an overpowering nausea, covered with a profuse perspiration. His rheumatism was all gone, the pain and redness had disappeared from the joints, and nothing remained except the extreme postration. Some broth was prepared for him, a little brandy given, a powder of calomel and morphia administered. Calomel—for at that time it was the regulation dose for irritable stomach or ingrowing toe nail; it would have been heterodoxy not to be thought of, to have omitted it.

The man rallied, and in a little time recovered. The after treatment was a bottle of bitters, made of a decoction of cimicifuga and gentian root, a pint, to which was added half a pint of whisky. Dose, a tablespoonful three times a day. Directions written on a half sheet of paper and tied to the bottle.

Here was a new idea in regard to rheumatism ; a direct refutation of all my teaching. "Gruel, warm covering and *six weeks*," "calomel and opium," "digitalis and alkalies," "corn sweets and calamus," and with all or any, four or six weeks. It made an impression on me, and it was not long before I had a case, a child of ten years, unmistakably affected. Wine of colchicum was the form chosen, given with care, in simple syrup, m. ij. every hour ; an opiate was first given, to mitigate the pain. Violent vomiting and purging came on at the fourth hour, and further medication had to be suspended ; there was no alleviation of the disease, and the expectant method was followed for three or four weeks, until the disease ran its limit, or nature overcame it. The term "self-limited disease" has done much injury to the practice of medicine, and the method has been fitly named, a "meditation on death." If medicine is of any value, it is in cutting short or modifying disease, and with so many articles of known power, to sit down and let nature wrestle with disease unaided, finds its best similitude in aboriginal surgery, that lets gangrene and inflammation amputate the injured arm in long months, while the knife of the surgeon could do is with much greater safety in a few minutes.

The next case called to was that of a delicate woman of thirty. Her limbs were swollen, the pain excruciating ; it was a third or fourth attack. There was evidence of valvular disease of the heart. Here was a case to treat carefully yet promptly. One-fourth grain doses of morphine were given, to mitigate the pain, repeated every hour until relieved. A prescription was ordered, consisting of vin. colchici. rad., m. x.; tinct. opii. m. x.; tinct. cardam. comp., q. s. to make up a teaspoonful, which was the dose, to be administered every three hours. This was continued for three days, alternating with the morphia. There was no nausea or movement of the bowels. The pain was quited and the heart kept steady; at the end of that time vomiting and purging came on together ; there was severe postration, requiring careful treatment for a few days, but the disease was conquered, and in a few days more the patient was discharged cured.

I have seen many cases since, and have tried all the various remedies used, but none with so much certainty or success as with colchicum. Combine it so that the stomach will retain it long enough for the system to become saturated with it. Give hypodermic injections of morphine to quiet the stomach and ease the pain, then add opiates and aromatics or alkalies, as the cases may be. If it has time to permeate the blood it acts as an antidote to the *materies morbi* that constitutes the disease, then come the specific action, vomiting and purging, and the end of the disease. If this result comes too soon no cure is effected. Sometimes we cannot so guard or disguise it that it will not be promptly rejected, and we get no benefit from it. In such cases we resort to the salicylic acid, which sometimes gives prompt relief, but with a tendency to relapse; at other times seems of little value. Then we try the alkaline treatment and patience. So far I have never failed with colchicum if it could be tolerated for two or three days. I have not hesitated to use it with children or feeble adults, or where there existed cardiac complications, and in those cases treated primarily, have seldom, if ever, had the disease affect the heart. I have also reason to think that in small doses it may act as a prophylactic in those cases that are liable to recur. In a case where lithic acid was deposited in large quantities by the urine, especially where the concretions were so large as to cause nephritic colic, it seemed to prevent the concretions, or to dissipate them. If acute rheumatism depends, as some think, on the accumulation of lithic acid, or its rapid formation, colchicum may serve to excite the system to expel it, and prevent its deposit. Vomiting and purging is not effectual if produced by other drugs, or even by colchicum, if it comes too soon.

I have in some instances used salicylic acid, or salicylate of soda, in from five to ten grain doses in the beginning of the case, in connection with the colchicum, which acted as a palliative: also chloroform liniments, warm applications and blisters, in severe cases, but only for immediate relief, while waiting for the saturation of the system with the drug which effects the cure.

In looking over authorities, I find a diversity of opinion expressed, the older authors holding that it was a valuable remedy for rheumatism; the later ones that it is uncertain, severe and of doubtful efficacy; all agreeing that it is a specific in gout. Bartholow says of it: "In medicinal doses it increases the mucus and glandular secretions of the stomach and intestines, and probably, also, of the liver and kidneys, and skin. In large doses, it produces a feeling of epigastric heat, nausea and vomiting, depression of the circulation, muscular feebleness, headache. It frequently purges, producing copious watery stools, and is usually held to promote the discharge of biliary matter. It increases the flow of the urine, of the solid constituents (urea, uric acid, etc.), as well as the water."

Dr. Austin Flint says, of rheumatism: "The majority of pathologists have adopted the supposition of Prout, that the morbid principle is lactic acid, and it is generally accepted as a rational base of treatment.

Fuller, in his elaborate work on the disease, claims for the alkaline treatment the arrest of the lactic acid formation and its elimination, and thus a speedy cure for the complaint. He considers the patient safe from cardiac complication when the urine becomes alkaline, and remains safe so long as this condition of the urine is maintained.

Flint says that, "Rationally, according to this view, what is most to be desired in the treatment is the arrest of the production of the *materies morbi* contained in the blood." I am of the opinion that the slow and cautious saturation of the system with colchicum does this, and that the vomiting and purging eliminates the urates already generated. I have resorted to the alkaline treatment with some success in those cases where the colchicum was too promptly rejected to be of any service, and latterly to the salicylic acid, but only where colchicum failed.

Quinine is a necessary adjuvant in malarial regions, but is of no avail alone; so also iodide of potassium is of advantage where there may be a syphilitic taint. This for acute rheumatism. The remedies and treatment of the chronic form is to be considered at another meeting.

